# Integrative Nanotechnology and Biomaterial Approaches for Targeted Diabetic Wound Regeneration

**DOI:** 10.2174/0115733998416898251230195238

**Published:** 2026-03-30

**Authors:** Arindam Kolay, Ravindra Pal Singh, Swetaleena Shaw, Aman Kumar, Sourabh Kosey, Shivam Singh

**Affiliations:** 1 Department of Pharmaceutics, NIMS Institute of Pharmacy, NIMS University, Rajasthan, Jaipur, India;; 2 Department of Pharmacy Practice, NIMS Institute of Pharmacy, NIMS University, Rajasthan, Jaipur, India;; 3 Department of Pharmacology, NIMS Institute of Pharmacy, NIMS University, Rajasthan, Jaipur, India

**Keywords:** Diabetic wound, hyperglycemia, nanoparticles, nanocarriers, biopolymers, scaffolds, regenerative medicine

## Abstract

Recent advancements in drug delivery technologies, bioactive molecules, and biomaterial design have transformed the therapeutic landscape of diabetic wound management. Diabetic wounds remain a major clinical challenge due to persistent inflammation, impaired angiogenesis, and reduced tissue regeneration, primarily caused by chronic hyperglycemia and oxidative stress. Emerging nanotechnology-based systems, such as polymeric, metallic, and lipid nanoparticles, enable site-specific drug delivery with enhanced stability, biocompatibility, and sustained release, thereby improving therapeutic efficacy. Complementary nanocarrier platforms like liposomes, niosomes, hydrogels, and nanohydrogels provide a moist microenvironment essential for cell proliferation and tissue remodeling while ensuring controlled drug release for faster wound closure. Advanced strategies, including dendrimers, transferosomes, microneedle arrays, and 3D-printed scaffolds, offer personalized, minimally invasive, and adaptive therapeutic solutions. Nanoemulsions and nanocapsules facilitate effective encapsulation and transport of hydrophobic drugs, broadening their clinical applicability. Incorporating bioactive growth factors such as VEGF, PDGF, chemokines, and stem cell-derived exosomes into these systems further enhances angiogenesis, modulates inflammation, and accelerates granulation tissue formation. Meanwhile, biopolymers like collagen, chitosan, hyaluronic acid, and silk sericin serve as natural scaffolds that support cell adhesion, migration, and epithelialization. Collectively, these emerging technologies signify a paradigm shift toward integrated, patient-specific, and regenerative approaches for diabetic wound healing, addressing the limitations of conventional therapies through the synergy of nanotechnology and bioengineering.

## 1 INTRODUCTION

Hyperglycemia is a hallmark of Diabetes Mellitus (DM), a “syndrome X” that includes both type 1 Diabetes Mellitus (T1DM) and type 2 Diabetes Mellitus (T2DM). This illness, which poses a serious worldwide health concern, is due to the deficiencies in either insulin secretion or insulin responsiveness [1, 2]. By 2045, an estimated 700 million people will have diabetes, according to the International Diabetes Federation data [3]. DM is among the most common severe illnesses worldwide, typified by hyperglycemia, which leads to major harm to the various organs of the body. The number of persons having DM is predicted to increase significantly in thecoming 10 years, based on2019 International Diabetes Federation (IDF) projections [4]. Almost 15% of people with diabetes may get Diabetic Wounds (DW) at some point in their lives. DW can be caused by either type 1 or type 2 diabetes. DWs usually appear, especially on the feet, and in extreme situations, they might result in amputations [5, 6]. Because DM impairs metabolic activity, it raises the chances of contamination, resulting in lower wound repair. Diabetic Foot Ulcers (DFUs), a chronic lesion that alters a patient's lifestyle and hence increases the risk of death, are complications that can arise as diabetes mellitus progresses [7]. Every year, between 9.1 and 26.1 million DM patients worldwide develop DFU. Patients with DM have a 25% probability of developing DFU, and regrettably, in many cases, the only viable treatment option is amputation. Due to infection and unresolved vascular damage, the mean three-year survival rate of DFU amputees is 50%. On the other hand, 50% to 70% of patients who have healed DFU after therapy may experience recurrence within five years [7, 8]. Numerous alterations and difficulties may result from the colonization of foot wounds, particularly when severe limb ischemia is present. These alterations frequently include foot dysfunction, elevated anxiety, dry skin, and impairments in motor and sympathetic functions [9, 10]. Diabetic foot ulcers are likely to occur in 15-25% of diabetic patients during their lifetime, with a 48% risk [11]. Recent advances in nanotechnology-based drug delivery systems and biomaterial engineering have opened new possibilities in diabetic wound management. Nanocarriers such as polymeric nanoparticles, liposomes, niosomes, and nanohydrogels enable site-specific, controlled, and sustained drug release, maintaining a moist microenvironment favorable for tissue regeneration. Furthermore, smart systems, such as dendrimers, transferosomes, and microneedle arrays, offer minimally invasive and customizable treatment options that enhance drug penetration and patient compliance [12].

In parallel, bioactive molecules including Vascular Endothelial Growth Factor (VEGF), Platelet-Derived Growth Factor (PDGF), stem cell exosomes, and anti-inflammatory cytokines are being incorporated into advanced delivery systems to accelerate angiogenesis, modulate inflammation, and stimulate cellular proliferation [13]. Complementary biomaterials like collagen, chitosan, hyaluronic acid, and silk sericin act as scaffolds that support cellular adhesion and epithelialization while maintaining structural integrity [14].

### 1.1 Classification of Acute and Chronic Wounds

Acute wounds, usually brought on by trauma or surgery, heal according to the regular phases and provide a predictable, ordered, and time-dependent pattern of tissue repair [15]. A pathologic process is often the source of chronic wounds, which do not heal as quickly as acute wounds and usually require medical intervention. Pressure ulcers, diabetic ulcers, and vascular ulcers are the three types of chronic wounds [16, 17]. Chronic wounds are metabolic disorders characterized by persistent hyperglycemia [18, 19] brought on by unmanaged glycemia and delayed by both large vessels and microvascular issues. Because of this, chronic wounds yield an extended time to repair than normal [20, 21].

Chronic wounds that do not heal are characterized by ongoing inflammation. It is possible that these nonhealing lesions remain for a longer time in inflammatory form and do not get better [22]. Recurrent tissue damage causes bacteria, especially biofilms, and proteins obtained from platelets, including TGF-β or molecules of ECM fragments, to increase the overabundance of inflammatory cells in the injured bed. The pro-inflammatory cytokine cascade, which comprises IL-1β and TNFα, is increased and persists for a long period, which causes the lesion's protease levels to grow. In acute wounds, proteases are tightly regulated by their inhibitors. Chronic wounds have larger quantities of proteases than their corresponding inhibitors, which disrupts the extracellular matrix and breaks down growth factors and the locations where they bind. Proteases break down Extracellular Matrix (ECM), which not only prevents the wound from entering the development stage but also attracts more inflammatory cells, thereby escalating the inflammatory cycle [23]. The phenotypic anomalies of the cells in the wound bed that originate from the epidermis and dermis are another characteristic of chronic wounds. These anomalies include decreased mitogenic capacity and growth factor receptor density, which hinder the resident cells' ability to react appropriately to signals for wound healing [24, 25].

### 1.2 Wound Healing Phase

#### 1.2.1 Normal Wound Healing

The progression of wound healing includes a number of unique and complex phases that can be interrupted by both local and systemic variables, including age, nutritional condition, moisture, contagion, maceration, and somatotype (systemic). As seen below, the entire procedure is traditionally divided into four successive steps (Fig. **1**) [26, 27].

A. Phase I: Hemostasis

B. Phase II: Inflammation

C. Phase III: Proliferation

D. Phase IV: Maturation and Remodelling

##### 1.2.1.1 Phase I: Hemostasis

Vascular constriction brought on by the sympathetic nervous system limits the drainage at this point, and it eventually begins to clot. The goal of hemostasis is to stop blood loss after an injury. The body starts its blood coagulation and crisis repair systems at this phase, building an obstacle to contain what remains. In order to safely gather cells at the injury site, damaged tissue cells emit maturation factors, chemical messengers, and warning signals that filter fragile cells from blood flow and promote the expansion of tissue-resident groups [28].

##### 1.2.1.2 Phase II: Inflammation

This category addresses coagulation, and the subsequent defensive/inflammatory phase seeks to clear the wounded area and bacteria to create room for the development of newly formed tissue. WBCs approach the wound during this stage to eliminate debris and kill microbes. The remaining macrophage cells among the WBCs continue to remove debris. The network of these cells is responsible for development and nutrition suppliers, as well as those that aid in the repair of damaged tissue. Erythema, heat, edema, and pain are the hallmarks of this four- to six-day period, which is considered chronic inflammation [29, 30].

##### 1.2.1.3 Phase III: Proliferation

Following the removal of the damage, the wound enters the third phase, during which the restoration and coverage are thought to be the primary targets. During the epithelialization phase, the granulation structure, which includes fibroblasts, smoothcells, and newly formed vasculature, allows skin cells to travel on top of this tissue, indicating the proliferation stage [31]. Proliferative stages include (1) wound filling, (2) wound region reduction, and (3) the development of a shrill sheet on the wound (epithelialization). Each species has a particular skin structure, which promotes rapid healing [32]. Typically, the proliferative period lasts four to twenty-four days.

##### 1.2.1.4 Phase IV: Maturation and Remodeling

Newly formed tissue progressively becomes stronger and more flexible during the maturation period. At this stage, collagen fibers reorganize the tissue as it heals and grows, increasing its overall durability (albeit its maximum strength is only 80% of its pre-injury strength). The maturity phase, which ranges from two weeks to a couple of years, varies greatly throughout the various wounds. Enhancement of other environmental factors that also occur during this period, such as “fibroblasts, fibronectin, and glycosaminoglycan” [33]. Collagen I (80-90%) and III (10-20%) create healthy skin, but 10% of developed scars include collagen III. Phagocytosis is facilitated by the overlap of collagen III and fibronectin [34, 35].

#### 1.2.2 Diabetic Wound Healing

The injured tissue will commence acute wound healing under normal physiological conditions [32, 36]. If microbial infection occurs or the fundamental pathogenic process impedes the healing process, the wound fails to heal and becomes chronic [37]. The main cause of diabetic wound damage is hypoxia, which is caused by two factors: an inadequate oxygen supply and high oxygen consumption in the wound [38]. Neuropathy and vascular dysfunction in people with diabetes can reduce the amount of oxygen that can reach the wound. In addition to insufficient oxygen delivery, wound hypoxia is also caused by excessive oxygen consumption by inflammatory wound cells [38]. A disparity among angiostatic (such as thrombospondins, endostatin, and angiostatin) and angiogenic (such as converting growth factor-β, TGF-β, fibroblast development factor 2, FGF2, vascular endothelial growth factor, or VEGF, and angiopoietins) factors may exacerbate wound hypoxia in diabetic patients [39, 40]. Furthermore, tissue remodeling is hampered by chronic inflammation, which is typified by an overabundance of M1 macrophages [41], decreased fibroblast proliferation and differentiation, and inhibited collagen production [42]. Through mechanisms such as MMP-9 overexpression, which breaks down collagen and hinders keratinocyte migration [43, 44], lowers VEGF and HIF-1Δ03b1 activity, and causes non-enzymatic glycation of proteins, hyperglycemia further exacerbates the problem [45]. Conventional treatments, such as wound care, infection management, and metabolic regulation [46], are meant to control symptoms, but they are frequently ineffective and protracted, leading to physiological stress and secondary injuries. To address the complications of diabetic wound healing and improve patient results, emerging advanced therapies with regulated release properties, such as topical medications, growth factors, cell-based treatments (*e.g.*, stem cell and fibroblast), and biomaterial-based approaches [47, 48], hold great promise.

## 2 MATERIALS USED IN DIABETIC WOUND HEALING

There are two distinct materials used to treat chronic wounds: one is “scaffolding materials”, the second is “temporary dressings”.

The best way of maintaining bodily fluid balance is by protecting the wound by allowing oxygen to enter, which stops environmental infections from proliferating without affecting the healing process [49]. To prevent issues during their removal, the materials used should be compatible with our immune system, non-biodegradable, and should not promote cell adhesion. Drug delivery and activity preservation are the two main purposes of dressings, which enable the medications to be released at the appropriate rate. The production of exudates, which include inflammatory cytokines and chemokines that encourage bacterial development, plays a crucial role in wound dressings [50].

### 2.1 Scaffolding Materials

To treat chronic wounds, scaffolding materials should encourage quick healing, restore function, and assist tissue regeneration, all while avoiding chronicity [51]. These substances must break down at a pace that corresponds to tissue expansion without causing toxicity or immunogenicity [51]. To avoid detachment or fracture during the healing process, adequate adherence to surrounding tissues and mechanical characteristics that mirror native skin are crucial. They should have a restricted capacity for swelling, maintain their water content, or use techniques to prevent dehydration and hold their form over time. Growth factors and medications should be transported locally at the healing site using scaffolds. Promising developments include engineered skin replacements that are made using 3D designs to resemble the extracellular matrix (ECM) and the natural cell environment [52]. These structures may be made especially well using biomimetic materials, which provide gas fluid permeability to absorb smells, keep the environment moist, and stop exudate buildup that might cause necrotic tissue development [50].

### 2.2 Materials in Temporary Dressings

Gauze, thin film, foam, hydrogels, and hydrocolloid membranes are among the several kinds of wound dressing treatments that are appropriate for treating particular kinds of wounds.

#### 2.2.1 Medical Gauze

Used extensively in wound treatment, it is composed of synthetic or natural fibers, such as polyester or cotton. It may be sanitized and absorbs exudates while keeping the wound moist. Additionally, to increase its adaptability and efficacy, gauze can be improved with additives such as saline, petroleum, antibiotics, or antiseptics [53].

#### 2.2.2 Dressing with Thin Film

Thin film dressings are translucent, artificial, flexible wound dressings composed of polyurethane. Because of its suppleness, the afflicted body component may move comfortably. It works as a semi-permeable material and is translucent, which facilitates oxygen exchange [54].

#### 2.2.3 Foams

Fabricated polymer foams, such as polyesters, may absorb more exudate than thin film dressings and have mechanical flexibility that allows the injured body part to move [55]. Additionally, it facilitates gas exchange and keeps the atmosphere wet [56].

#### 2.2.4 Hydrogels

Amorphous, sheet, or impregnated gauze forms are available for hydrogels, which are 3D crosslinked structures that absorb a lot of water [55, 57]. They are simple to apply and remove, and they provide moisture to dry or hardly exuding wounds. However, they have drawbacks, such as gas and oxygen permeability, which lessen their capacity to fight infections. Antibacterial hydrogels, including chitosan containing ZnO nanoparticles, have been created to improve wound healing [58].

#### 2.2.5 Hydrocolloid

These colloidal materials, which are frequently referred to as gel-forming agents, are typically made from pectin, gelatin, or carboxymethylcellulose [59]. The colloidal components engross the exudate when applied topically to wounds, forming a gel-like material that adheres to the wounds while letting water and oxygen through. It provides easy removal, thermal insulation, and a moist environment [60].

## 3 FACTORS AFFECTING WOUND HEALING

Studies reveal that the normal healing mechanism seen in acute wounds differs greatly from the healing procedure that occurs in chronic diabetic wounds.

### 3.1 Vasculopathy

Type 1 diabetes causes microvascular complications, impairing blood flow and oxygen delivery to wound sites. Unlike non-diabetic individuals, diabetic patients experience sluggish distal vein function, particularly in the femoral, tarsal, and metatarsal veins, which disrupts the supply of oxygen and essential nutrients required for wound repair [61, 62].

### 3.2 Neuropathy

Patients with diabetes lose sensorimotor and autonomic nerve fibers, which impairs their perception of pressure, temperature, and other environmental stimuli. Patients are unable to identify injuries due to this lack of sensation, which makes wound healing more challenging. Additionally, altered vasodilatory autoreceptors, which reduce pain perception, hinder the healing process and contribute to wound formation by impairing the body’s ability to respond to force and injury [63].

### 3.3 Infection

A major contributing cause to delayed wound healing is infection, which frequently results in consequences including limited mobility, hospitalization, and extended recovery periods. Diabetes wounds, especially ulcers and abrasions, are more susceptible to infection, which might result in inflammation, cellulitis, and osteomyelitis, all of which require prompt medical attention [64, 65].

## 4 MANAGEMENT

### 4.1 Biomaterial Agents

Biomaterials are non-pharmaceutical substances that are utilized to enhance organs, tissues, and cells. These are made of artificial polymers derived from plants and aid in wound healing. A list of biomaterial agents for diabetic wound healing is written below in Table **1** [66-79].

### 4.2 Bioactive Agents for Wound Healing

Bioactive drugs, which are commonly utilized to improve diabetic wounds, comprise both non-functionalized and functionalized compounds.

#### 4.2.1 Functional Molecules

##### 4.2.1.1 Signaling Molecule

Molecules that Signal Cellular processes depend on natural lipids such as phospholipids and sphingolipids [80]. By attracting inflammatory and Mesenchymal Stem Cells (MSCs) to promote tissue restoration, chemokines are essential for angiogenesis, extracellular matrix remodeling, and epithelial formation again [81]. By coordinating cellular connections throughout the wound-healing procedure, the combination injection of bFGF and VEGF speeds up vascular regeneration and aids in muscle recovery [82, 83].

##### 4.2.1.2 miRNA, Gene, Protein, and Peptide

miRNA, also referred to as miR-26a, is detected in diabetic wounds. By limiting this miRNA, diabetic wounds can be treated more effectively. miRNA can cure different characteristics and cycles because of its pleiotropic effect [84]. Drug delivery systems of genes, proteins, or peptides are listed in Table **2** [85-94].

##### 4.2.1.3 Stem Cell / Exosomes

Layered vesicles at the nanoscale are called exosomes. To improve tissue repair and restore blood flow, collagen hydrogel is combined with Adipose-Derived Stem Cells (ADSCs) and Epidermal Growth Factor (EGF) microcapsules [95, 96]. A few loading nanomaterials for DWH are mentioned below in Table **3** [97-105].

##### 4.2.1.4 Natural and Synthetic Drug Delivery Systems

Drugs along with DDS and their action of natural and synthetic delivery systems are listed below in Table **4** [106-119].

#### 4.2.2 Non-functional Molecule

##### 4.2.2.1 Metal Ion

These metallic ions include various types that are detailed in Table **5** below, but silver nanoparticles, namely AgNP, are thought to be quite useful among them. They have antibacterial, calming, and wound-healing capabilities. List of metal ions with their route of administration is attached below in Table **5** [120-127].

### 4.3 Novel Drug Delivery System (NDDS)

Numerous innovative medication delivery approaches have been investigated for diabetic patients' wound management.

#### 4.3.1 Nanoparticles (NPs)

Steel and metal oxide nanoparticles, as well as non-metallic nanomaterials, are now found in NPs used to treat diabetic wounds. NPs are adaptable carriers made for precise and regulated medication administration. Their nanosize improves therapeutic effectiveness by facilitating effective cellular absorption [128].

##### 4.3.1.1 Polymeric Nanoparticles (PNPs)

Made up of biodegradable substances such as Polylactic Acid (PLA), chitosan, and poly (lactic-co-glycolic acid) [129]. PNPs can effectively transport the active medication to the desired location and are reasonably priced [130-132]. These work especially well for stable and long-lasting medication administration. By promoting tissue regeneration, chitosan nanoparticles improve wound healing and have antibacterial qualities [4, 133].

##### 4.3.1.2 Solid Lipid Nanoparticles (SLN)

These are constructed out of useful polymers, replaced with liposomes as medical assistances, and become available as potent water-soluble remedies. They can decrease the lethality of drugs and improve their portability. SLNs are highly desirable because of their capacity to advance the distribution of prescribed drugs, and they have unique characteristics like high drug loading, large surface area, and short length [134].

##### 4.3.1.3 Nanostructured Lipid Carriers (NLC)

Partially crystalline oil particles are distributed in an aqueous portion that contains an emulsifier to create Nanostructured Lipid Carriers (NLCs), which improve drug loading, encapsulation performance, and stability, as well as drug bioavailability, bioactive substance balance, and shelf life [135].

##### 4.3.1.4 Microemulsion (ME)

These are water-in-oil (W/O), which is made up of an aqueous phase that is spread over an oily area. It facilitates regulated release, improves medication delivery, and aids in wound healing [136].

##### 4.3.1.5 Nano-Emulsion (NE)

A surfactant can be added to NE to stabilize the two immiscible liquid components it contains. When nanoemulsions are given topically to wounds, the emulsions penetrate the epidermis and heal the wound. Compared to microemulsions, nanoemulsions are more capable of lipophilically loading drugs [137]. Essential oil, which makes up NEs, has a stronger antimicrobial impact when administered topically [138].

##### 4.3.1.6 Nanosuspension (NS)

Medicaments in the aqueous phase are made up of a non-aqueous or aqueous medium steadied by polymeric or surfactant stabilizers. A promising method addressing solubility issues, the nanosuspension era offers several benefits, such as improved oral bioavailability and dissolution [139].

##### 4.3.1.7 Nanocapsules

The primary component of nanocapsules is a liquid/solid mixture, with the active medication embedded in the cavity and protected by a membrane made of polymers. When it comes to intracellular and target-based wound care, polymeric nanocapsules work better [140, 141].

#### 4.3.2 Liposomes and Niosomes

##### 4.3.2.1 Liposome

Both hydrophilic and lipophilic medications are encapsulated in spherical vesicles called liposomes, which have a phospholipid bilayer [142]. They lessen systemic toxicity by offering biocompatibility and regulated release. By administering growth factors, antibiotics, or anti-inflammatory drugs, they successfully cure wounds [143, 144]. According to a study, di-hydro quercetin-loaded liposomes exhibit enhanced antioxidant properties and offer greater potential for skin wound healing [145].

##### 4.3.2.2 Niosomes

Like liposomes, but made of non-ionic surfactants that trapped both hydrophilic and hydrophobic molecules together [146, 147], niosomes are less expensive and more chemically stable. A variety of medicinal drugs can be delivered with them, and they improve drug permeability [148].

#### 4.3.3 Hydrogels and Nanohydrogels

##### 4.3.3.1 Hydrogels

These are 3D, water-loving polymerous webs skilled at capturing considerable wound exudates while providing a moist healing environment. They have a soft consistency and a high porosity. Both “gram +ve” and “gram -ve” bacteria, and other bioactive compounds, are susceptible to the antimicrobial effects of hydrogels, which make them good transporters of growth factors and antibiotics [149, 150].

##### 4.3.3.2 Nanohydrogels

They provide better drug retention, regulated hydration, and increased *in vivo* compatibility as a nanoscale variant of hydrogels [151]. Because they prolong hydration and promote tissue regeneration, they are especially helpful in diabetic wounds [152]. Gellan-cholestrol was introduced to promote a rapid wound recovery [153].

#### 4.3.4 Dendrimers

The tree-like, highly branching polymers known as dendrimers have a large surface area that is ideal for drug conjugation. They make minimum toxicity, controlled release, and precise targeting possible. Growth factors and anti-inflammatory drugs are being investigated for direct delivery to wound sites using dendrimers [154].

#### 4.3.5 Transferosomes

These are ultra-flexible vesicles capable of entering deeper layers of the skin. These vesicles are perfect for burns and chronic wounds because they efficiently transfer medications to the underlying tissues. Improved transdermal delivery of bioactive compounds has been achieved through the use of transferosomes [155].

#### 4.3.6 Microneedles

Drug delivery through the skin with microneedles is a minimally invasive procedure that does not cause a lot of discomfort or harm. Microneedle patches filled with insulin or antibiotics have shown potential in healing diabetic wounds, improving glucose management, and lowering infection risk.

#### 4.3.7 Nanofibers

Electrospun nanofibers encourage cell attachment and proliferation by imitating the extracellular matrix. These fibers are useful for wound dressing applications because they may be loaded with medications such as growth hormones or antibiotics for prolonged release [156, 157]. When used to fabricate NFs, Thermally-Induced Phase Separation (TIPS) demonstrated increased water vapor absorptivity and maintained the rate of active drug release [158].

#### 4.3.8 Polymeric Micelles

In preclinical research on diabetic wounds, they are frequently utilized. Polymers in superficially accurate compositions are used to create them. In order to restore wounds in diabetic rats, several polymer groupings for maximal loading, consistency, systemic circulation, and distribution may be investigated, thanks to the accessibility of hydrophobic and hydrophilic polymer matrices [159, 160].

## 5 ADVANCED DRUG DELIVERY SYSTEM

### 5.1 Photothermal Therapy (PTT)

Near-Infrared (NIR) lasers and light-absorbing NPs are working in Photothermal Therapy (PTT), a common cancer treatment method, to create localized high temperatures surrounding Nanoparticles (NPs) that cause cell death. Recent developments include:

#### 5.1.1 Nanoparticles of BSA-CuS

These nanoparticles, which were created by a biomineralization process with Copper Sulfide (CuS) and Bovine Serum Albumin (BSA), show potent antibacterial properties when exposed to near-infrared light [161, 162].

#### 5.1.2 PB@PDA@Ag Nanoparticles

Under NIR, PB@PDA@Ag nanoparticles demonstrate effectiveness in hastening the healing of diabetic wounds [163].

#### 5.1.3 MoS2-BNN6 Nanocomposites

*Staphylococcus aureus*, heat-resistant *Escherichia faecalis*, ampicillin-resistant *Escherichia coli*, and other resistant bacteria are successfully inhibited from growing by MoS2-BNN6 nanocomposites [164]. PTT can cure diabetic wound infections, but it also has the potential to harm nearby healthy tissues; more thorough studies and clinical trials are required.

### 5.2 Layer-by-Layer Self-Assembly Technique

This method is frequently used to improve drug transport and material characteristics [165, 166]. To improve drug release, stability, mechanical characteristics, and hydrophilicity, this approach alternatively deposits layers with opposing charges onto polyelectrolyte matrices [167, 168]. It is simple, controllable, economical, and minimizes complications, making it a preferred method [169].

#### 5.2.1 Applications

Because of its poor integration, Natural Rubber Latex (NRL) is only used externally or for brief periods to treat chronic wounds. The performance and speed of membrane synthesis can be improved by LBL technology [170, 171], which includes:

#### 5.2.2 Therapeutic Coating

siRNA and other therapeutic agents may be released under regulated conditions thanks to nanoscale LBL coating, which speeds up the healing of diabetic wounds [172].

#### 5.2.3 Implications

LBL can alter localized protein expression, which may help cure problems including cancer, transplant rejection, Diabetic Foot Ulcers (DFUs), and cardiovascular disorders.

#### 5.2.4 Biomedical

LBL is a useful instrument for biomedical breakthroughs due to its adaptability and efficiency.

### 5.3 3D-Printed

Significant benefits of three-dimensional-printed scaffolds for wound dressings include configurable dimensions (*e.g.*, area, thickness, pore size), improved oxygen permeability through porous designs, simplicity of medication loading, and material diversity [173].

#### 5.3.1 Sodium Alginate/Polyethylene Glycol (SA/PEG) Scaffolds

Using 3D printing, SA/PEG scaffolds were produced with dissimilar PEG concentrations (1-5%). These scaffolds showed potent antibacterial action, especially against gram-positive bacteria. Both on and inside the scaffold, their porosity and characteristics promote cell development [174].

#### 5.3.2 Microneedle Patch Systems

To alleviate diabetes, microneedle patches for negligibly aggressive, glucose-responsive insulin administration were developed using post-stretching and extrusion-based 3D printing [175].

#### 5.3.3 Negative Pressure Wound Therapy (NPWT)

NPWT is a cutting-edge method that is frequently effective for complex surgical wounds and diabetic patients. To encourage healing and decrease wound volume, it exerts negative pressure, eliminates exudates, maximizes blood flow, and keeps the wound environment wet [176]. It entails delivering regulated negative pressure to the wound region while attaching a vacuum source to a wound filler. A computer-controlled retrograde installation is a recent development that allows for the repeated administration of an antibiotic or antiseptic solution with saline to the wounds. By facilitating the targeted delivery of advantageous chemicals straight to the wound sites, this improvement raises the therapeutic potential of NPWT (Table **6**) [177, 178].

## 6 DISCUSSION

The major reason diabetic wound healing is a complicated and drawn-out process is that long-term hyperglycemia disrupts critical processes, including angiogenesis, inflammation resolution, and extracellular matrix remodeling. These wounds have a major impact on patients' quality of life and come with high medical expenses because of their chronic inflammation, poor neovascularization, and increased risk of infection. Hemostasis, inflammation, proliferation, and remodeling are the four overlapping stages of wound healing. In the inflammatory phase, immune cells are recruited to remove debris, while hemostasis starts the creation of clots and the release of growth factors. Persistent inflammation in diabetes postpones the fibroblast and endothelial cell formation of granulation tissue and blood vessels during the proliferation phase. Angiogenesis and epithelialization are further decreased by impaired VEGF activity. The majority of diabetes wounds are unstable, and this final stage of remodeling is not entirely completed. Numerous synthetic and natural materials aid in the care of diabetic wounds. Within these biopolymers, alginate, chitosan, collagen, and hyaluronic acid improved cell adherence, antibacterial activity, and moisturization. In contrast, the synthetic polymers PLGA and PEG are included because they offer a prolonged release profile of the therapeutic agents together with structural integrity. Electrospun nanofibers and advanced scaffolds can promote tissue regeneration because they biomimic the structure of the extracellular matrix. Because they offer prolonged medication release and site-specific treatment, novel drug delivery methods have completely changed wound care. The stability and bioavailability of medications are improved by nanoparticles, including lipid-based, metallic, and polymeric forms. A moist environment is maintained for tissue healing by hydrogels and nanohydrogels, while liposomes and Niosomes improve penetration and controlled release. While 3D printing scaffolds enable customization for particular wound demands, minimally invasive medication administration is made possible *via* dendrimers, transferosomes, and microneedle devices. The hydrophobic drug delivery provided by nanoemulsions and nanocapsules further expands the treatment choices. Through improved angiogenesis and tissue regeneration, these systems may be combined with a variety of bioactive substances, including VEGF, PDGF, chemokines, and exosomes from stem cells, which hasten the healing process of wounds. Collagen, hyaluronic acid, and silk sericin are examples of biomaterials that support cell adhesion and have a structural function. In summary, certain significant challenges in diabetic wound healing are resolved by combining bioactive chemicals, biomaterials, and sophisticated drug delivery systems. These cutting-edge technologies have really changed the game by opening the door to effective and individualized wound care solutions.

## CONCLUSION

Chronic hyperglycemia leads to decreased tissue regeneration, poor angiogenesis, and prolonged inflammation, all of which hinder the complex process of diabetic wound healing. To overcome these obstacles and enhance treatment results, creative approaches are required. With their capacity to provide tailored treatments, prolonged drug release, and improved bioavailability, bioactive compounds, biomaterials, and advanced drug delivery systems have become game-changing technologies. Polymeric, metallic, and lipid-based nanoparticles are among the many types of nanoparticles that are crucial for improving medication stability and delivery accuracy. Liposomes, niosomes, hydrogels, and nanohydrogels are examples of complementary systems that provide a regulated release and a moist environment at the wound site, which speeds up the healing process. New technologies, including 3D-printed scaffolds, dendrimers, transferosomes, and microneedle systems, have made it possible to treat complex wounds in a less intrusive and customized way. Additionally, by providing an efficient means of administering hydrophobic medications, nanoemulsions, and nanocapsules expand the range of available treatments. By encouraging angiogenesis, controlling inflammation, and inducing tissue regeneration, the addition of bioactive substances to these delivery systems, such as growth factors like VEGF and PDGF, chemokines, and exosomes derived from stem cells, will improve wound healing even more. Collagen, hyaluronic acid, chitosan, and silk sericin are examples of biomaterials that promote mechanical reinforcement, cell adhesion, and epithelialization. As a result, each of these many products will address the three main issues that diabetic wound healing presents: inflammation, infection, and disrupted tissue repair. With the utilization of cutting-edge medication delivery methods, active biological agents, and biomaterials, this sector has the potential to be greatly improved with further study. This will entail clinical trials and interdisciplinary research to improve these technologies and make individualized, practical, and affordable diabetic wound care treatments a reality.

## Figures and Tables

**Fig. (1):**
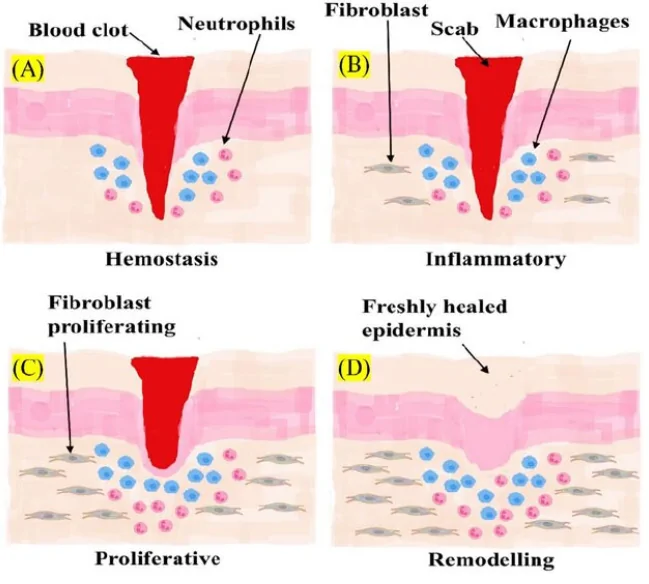
Stages of wound healing: (**A**) Hemostasis, (**B**) Inflammatory, (**C**) Proliferation, (**D**) Remodeling. Available online under the terms of the Creative Commons Attribution Non-Commercial License 4.0 [27].

**Table 1:** Biomaterial agents for diabetic wound healing.

**S.No.**	**Biomaterial agent**	**Biomaterials**	**Functions**	**Properties**	**References**
1.	Hyaluronic acid	Sponges, Membranes, Injectable Scaffolds, films, Hydrogel	The functions of hyaluronic acid include granulation tissue creation, inflammation, re-epithelization, proliferation, re-modelling, and scar formation.	Nonimmunogenic, biodegradable, having water-holding capacity and sorption.	[66, 67]
2.	Pectin	Film, membrane, film dressing, hydrogel	Improves ester’s secretion and galacturonic acid, anti-inflammatory and stimulates COX-2 and NOs enzymes.	Biodegradable, biocompatible polymer for wound repair.	[68, 69]
3.	Gelatine	Microcryogel, 3D scaffolds, hydrogels, and dressings.	On collagen hydrolysis, it holds more peptides and treats wound	Biodegradable, biocompatible, and non-immunogenic properties.	[70]
4.	Keratin	Scaffolds, dressing, transdermal patches, membrane, films.	Increases the activation of Schwann cells and functions similarly to gelatin.	Biodegradable, biocompatible, nontoxic.	[71, 72]
5.	Chondroitin sulfate	Nanofibers, transdermal patches, scaffolds.	Chitosan activates DWH and fibroblast production.	Least toxic, biodegradable, and biocompatible.	[73]
6.	Alginic acid	Injectable, sponges, hydro fiber, film membrane, dressing.	Cellular activation offers a moist wound climate.	Biocompatible, biodegradable, non-toxic.	[74]
7.	Collagen	Nanoparticles loaded hydrogel, 3D scaffolds, and Sponges.	Elevates the nutrition career, fibroblast, and accumulation of new collagen in the wounded area.	Biodegradable in nature and compatible, producing an inflammatory effect.	[75]
8.	Carboxymethyl cellulose	Sponges, Hydrogel.	Makes up for transdermal water loss.	Biodegradable and nontoxic.	[76]
9.	Fibrin and fibrinogen	3D printed structures, nanoparticles, bandages, scaffolds	Fibrin increases migration, proliferation, and adhesion of cell walls.	It has low toxicity, biocompatibility, and biodegradability.	[77]
10.	Silk sericin	Ointment dressings, scaffolds, film, *In-vivo*	Stimulates collagen development, also offers the propagation of osteoblasts, and keratinocytes.	Biocompatible, antioxidant potential, and immunological inertness.	[78, 79]

**Table 2:** Drug Delivery System of Genes/Proteins/Peptides with Nanocarriers.

**S.No.**	**Genes/Proteins/Peptides**	**Delivery System**	**Action**	**References**
1.	siRNA (downregulation of PHD-2)	AuNPs	Enhance siRNA's endosomal exit; silence PHD-2 in fibroblasts; and activate pro-angiogenic pathways.	[85]
2.	Keap1 siRNA	Lipoproteoplex (LPP) nanoparticle	Enhance the reduction-oxidation equilibrium in the wounded surroundings, speed up diabetic tissue regrowth, and recover Nrf2 antioxidant activity.	[86]
3.	Small interfering RNA-29a gene.	HA-PEI nanoparticles	Inhibit pro-inflammatory factors, speed up diabetic wound repair, and generate angiogenesis markers (α-SMA and CD31).	[87]
4.	MicroRNA (miRNA) medium interfering R-31-5p	Milk-extracted exosomes	Promote the propagation, migration, and angiogenesis of endothelial cells.	[88]
5.	Medium interfering R146a	Cerium oxide nanoparticles (CNP)	Scavenging free radicals, blocking the NFκB pathway, and reducing swelling.	[89]
6.	Antimicrobial peptide (LL37)	Ultra-small gold nanoparticles	Increase the efficacy of gene transport by improving the cellular and nuclear entry.	[90]
7.	Bioactive peptides	Chitosan NPs	Reducing the swelling phase and promoting new vascular formation.	[91]
8.	PDGF-BB proteins	Fibrin-based hydrogel	It promotes the formation of new blood vessels and increases the size of arterial blood vessels.	[92]
9.	VEGF-A mRNA	Ionizable lipid-mediated nanoparticles	Upregulate VEGF-A expression and advance healing development.	[93]
10.	Recombinant human thrombomodulin (rhTM)	NLC	Advances in injury curative and cell movement.	[94]

**Table 3:** Stem Cell/Exosomes Loading Nanomaterial for Diabetic Wounds.

**S.No.**	**Stem Cell/Exosomes**	**DDS**	**Action**	**Referenes**
1.	BMSCs	Nanofiber scaffolds, N-chitosan/HA-ALD hydrogel	Encourage collagen accumulation, gran formation, cellular development, vascular development, and the release of development factors through bone marrow mesenchymal stem cells (BM-MSCs) while controlling the inflammatory milieu.	[97-99]
2.	ADSCs	Hydrogels injection	Start the regeneration process; encourage the skin's collagen matrix, hair roots, and circulation to rebuild.	[100, 101]
3.	Gingival mesenchymal stem cells (GMSCs)	Silk hydrogel sponge	Collagen remodeling, epithelial regeneration, accumulation, and enhancement of revascularization and neuron growth.	[102]
4.	Mesenchymal stem cells (MSCs)	ADM-RGO fused scaffolds	Vascularization and collagen accumulation also lead to new epithelial formation	[103]
5.	Platelet-rich plasma exos	Chitosan/Silk hydrogel sponge	Vascularization and collagen deposition.	[104]
6.	MSC s	PLGA NPs	Induce capillary construction and collagen deposition.	[105]

**Table 4:** List of Natural and Synthetic Drug Delivery Systems.

**S.No.**	**Drugs**	**Delivery System**	**Action**	**References**
1.	Metformin hydrochloride	Niosomes, transdermal patches	Anti-diabetic effect and wound healing	[106]
2.	Mefenide acetate	Chitosomes, liposomes	Anti-bacterial activities promote wound healing	[107]
3.	Cephradine	Nanofibres	Helps in chronic inflammation	[108]
4.	eNos/nitrous oxide	Tissue engineering scaffolds	Skin wound healing	[109]
5.	Defer examine (DFO)	Hydrogels, or nanofiber scaffolds, transdermal	Upregulation VEGF	[110]
6.	Spiro-tetrahydroquinoline	NMR technology	Improves keratinocytes and wound-healing properties	[111]
7.	Quercetin	Scaffolds	inflammatory activity management	[112]
8.	Glycosaminoglycan	Polysaccharides	curing to the fullest	[113]
9.	Dimethyloxalyl Glycine (DMOG)	Porous electrospun fibrous membranes	Diabetic-impaired wounds	[114]
10.	Methylene blue	Niosomes	Effect of antioxidants on diabetic mice with wounds on their skin	[115]
11.	Ciprofloxacin	Cyclodextrin polymer sponges or chitosan	Wound healing	[116]
12.	Piper betel	Gel	Wound healing	[117]
13.	Curcumin	CS nanoparticles, gelatine, microsphere	Wound healing, anti-inflammatory, and anti-oxidant	[118]
14.	Piroxicam	Transdermal DDS	Anti-inflammatory	[119]

**Table 5:** Showing The List of Metal Ions with their Route of Administration.

**S.No.**	**Metal ions**	**Drug Delivery System**	**Route of Administration**	**Action**	**References**
1.	Gold (Au)	Human Peptide LL-37@Gold Nanoparticles (LL-37@AuNPs)	Topical and Oral	Diabetes wounds, whether or not they are infected by bacteria, can be effectively treated with a nano as well as with a novel quality conveyance framework.	[120]
2.	Ernest NAGEL particles	Nanofibers, scaffold, Gelatin	Topical	Releasing Si Particles, Effect on diabetic wound healing	[121]
3.	(Rb) Rubidium	Rb-containing Calcium Alginate gel, Hydrogel	Topical	Effective as an anti-inflammatory and shows the impact on the diabetic wound.	[122]
4.	Calcium (Ca) and Silicon (Si) ions	Bulgarian (BG) membrane and BG/Agarose-Alginate (AA) hydrogel.	Topical and Injectables	BG nanoparticles promote microphase advances to improve the wounded area's angiogenic state.	[123]
5.	Silver (Ag)	PB@PDA@Ag nano-composite, and hydrogel	Topical and Injectables	Dropping MRSA through NIR topical sensors.	[124]
6.	Copper (Cu)	HKUST-1 and BSA-CuS Nanoparticles	Oral and Topical	As atoms release NOs, copper reduces particle toxicity and cell death and enhances the management of bacterial infections.	[125]
7.	Molybdenum disulfide	Functionalized MoS_2_ nano-vesicles	Topical	By regulating the nitrous oxide emission, MoS2-BNN6 nanovesicles may produce nitrous oxidative pressure.	[126]
8.	Iron-Copper	Bi-metal Cu-Fe NPs	Topical	Germicide and damage healing activities.	[127]

**Table 6:** Comparative Summary of Advanced Drug Delivery Systems (DDS) for Diabetic Wound Healing.

**Drug Delivery System (DDS)**	**Mechanism of Action / Key Features**	**Advantages**	**Disadvantages / Limitations**	**Current Clinical Status / Examples**	**Future Prospects**
**Liposomes**	Phospholipid vesicles encapsulating hydrophilic and lipophilic drugs promote localized delivery and moisture retention.	Biocompatible, enhances drug stability, mimics biological membranes.	Physical instability, limited drug loading, and short shelf life.	Several wound-healing creams and ointments are in clinical use (*e.g.*, liposomal insulin gels).	Development of stimuli-responsive and targeted liposomes for chronic wounds.
**Polymeric Nanoparticles (NPs)**	Biodegradable polymers (PLGA, chitosan) provide controlled and sustained drug release.	Precise targeting, enhanced bioavailability, and protection of labile drugs.	Potential toxicity, scale-up difficulties, and complex regulatory approval.	Multiple preclinical studies on VEGF and antibiotic-loaded NPs.	AI-guided nanocarrier design and green synthesis for safe, scalable production.
**Metallic Nanoparticles (*e.g.*, Ag, Au)**	Exhibit antimicrobial and anti-inflammatory effects; can stimulate angiogenesis.	Strong antimicrobial activity, easy surface modification.	Cytotoxicity at high doses, oxidative stress, and non-biodegradability.	Used in antimicrobial dressings (*e.g.*, silver nanoparticle hydrogels).	Designing biocompatible metallic nanocomposites with reduced toxicity.
**Hydrogels**	Cross-linked polymeric networks that retain moisture and allow drug diffusion.	Excellent biocompatibility, provides a moist environment, and supports cell proliferation.	Poor mechanical strength, dehydration, and difficult sterilization.	FDA-approved wound dressings available (*e.g.*, collagen-chitosan hydrogels).	Smart, self-healing, and stimuli-responsive hydrogel formulations.
**Microneedle Systems**	Arrays of micro-projections for painless transdermal delivery of drugs or growth factors.	Minimally invasive, avoids first-pass metabolism, enhances penetration.	Limited drug loading, risk of microbial contamination.	Early clinical trials for insulin and peptide delivery.	Integration with sensors and biodegradable polymers for personalized therapy.
**Exosome-based Systems**	Natural nanovesicles delivering proteins, RNAs, and growth factors for tissue repair.	Bioactive, low immunogenicity, promotes angiogenesis and cell migration.	Isolation challenges, high production cost, and stability issues.	Preclinical models show improved diabetic wound closure.	Synthetic exosome mimetics and scalable bioreactor production.
**3D-Printed Scaffolds**	Biomimetic constructs supporting tissue regeneration and drug release.	Structural support, customizable geometry, mimics ECM.	High fabrication cost, limited biomaterial inks, and regulatory barriers.	Experimental stage; few preclinical successes with polymer-growth factor scaffolds.	Hybrid bioprinting combining smart nanocarriers and growth factor gradients.
**Nanoemulsions / Nanocapsules**	Nanodroplet systems enhancing solubility and absorption of hydrophobic drugs.	Stable, scalable, improves permeation and bioavailability.	Risk of phase separation, limited encapsulation of hydrophilic drugs.	Used in topical wound formulations.	-

## LIST OF ABBREVIATIONS

DM: Diabetes Mellitus
T1DM: Type 1 Diabetes Mellitus
T2DM: Type 2 Diabetes Mellitus
IDF: International Diabetes Federation
DW: Diabetic Wounds
DFUs: Diabetic Foot Ulcers
VEGF: Vascular Endothelial Growth Factor
PDGF: Platelet-Derived Growth Factor
ECM: Extracellular Matrix
